# Supplementary material for: HER2-Driven Breast Cancer: Role of the Chaperonin HSP90 in Modulating Response to Trastuzumab-Based Therapeutic Combinations
Source: Int J Mol Sci. 2025 Jul 9;26(14):6593. doi: 10.3390/ijms26146593 (PMC12294885; doi:10.3390/ijms26146593)
Supplement: Supplementary file 1 [file ijms-26-06593-s001.zip › ijms-3689162-supplementary.pdf]

Supplementary Figure S1. Influences of single trastuzumab and docetaxel treatments on cellular growth. (A) MCF7, ZR75-1 (HER2 negative), AU565 and BT474 (HER2 amplified) cells were treated with trastuzumab (Trast) at indicated concentrations for 72 and 96 hours. Cell viability was assessed by Crystal Violet assay and results, expressed as percentage of cell growth relative to untreated control, represent the average ±SEM of three independent experiments. **(B)** Cell lines described above, were treated with docetaxel (Doc) at indicated concentrations for 72 and 96 hours. Cell viability was assessed by Crystal Violet assay and results, expressed as percentage of cell growth relative to untreated control, represent the average ±SEM of three independent experiments.

Supplementary Figure S2. Stably HER2 transfection influences response to single and combination treatments and HSP90 expression (A, B) MCF7 cells were stably transfected with empty vector (MCF7-EV) or with HER2 plasmid constitutively active (MCF7-HER2) according to the manufacturer's protocol. HER2 levels were evaluated by Western Blotting using specific antibodies for HER2 and β-Actin was used as blotting control. MCF7-EV and MCF7-HER2 cells were treated with trastuzumab (Trast) and docetaxel (Doc), at indicated concentrations, for 72 hours. Cell viability was assessed by Crystal Violet assay and results, expressed as percentage of cell growth relative to untreated control, represent the average ±SEM of three independent experiments. Asterisks and symbols indicate statistically significant differences ($p < 0.05$ by 2-tailed Student's t test) for the comparison between *trastuzumab and combination-treated cells or §docetaxel and combination-treated cells. **(C)** MCF7-EV and MCF7-HER2 were treated with trastuzumab and docetaxel, alone or in combination, for 72 hours at indicated concentrations. Cells lysated were analyzed by Western Blotting using specific antibodies for HER2 and HSP90 and GAPDH was used as blotting control.

Supplementary Figure S3. HSP90 silencing influences response to trastuzumab and docetaxel combination treatment. MCF7-HER2 cells were transient transfected with a siRNA targeting human HSP90 for 72 hours, according to the manufacturer's protocol. HSP90 levels were evaluated by Western Blotting using specific antibodies for HSP90 and GAPDH was used as blotting control. Subsequently, the cells were treated at indicated concentrations with trastuzumab (Trast) and docetaxel (Doc), alone and in combination, for 72 hours. Cell viability was assessed by Crystal Violet assay and results, expressed as percentage of cell growth relative to untreated control, represent the average ±SEM of three independent experiments. Asterisks and symbols indicate statistically significant differences ($p<0.05$ by 2-tailed Student's t test) for the comparison between *trastuzumab and combination-treated cells.

Supplementary Figure S4. Stable inhibition of HSP90 or its activity modifies response to trastuzumab, docetaxel and their combination in HER2 context. (A) AU565 cells were stably transfected with a short hairpin (shRNA) against HSP90, according to manufacturer's protocol. HSP90 levels were evaluated by Western Blotting using specific antibodies for HSP90 and GAPDH was used as blotting control. Subsequently, the cells were treated at indicated concentrations with trastuzumab (Trast) and docetaxel (Doc), alone and in combination, for 72 hours. Cell viability was assessed by Crystal Violet assay and results, expressed as percentage of cell growth relative to untreated control, represent the average ±SEM of three independent experiments. Asterisks and symbols indicate statistically significant differences ($p < 0.05$ by 2-tailed Student's t test) for the comparison between *trastuzumab and combination-treated cells or or §docetaxel and combination-treated cells. **(B)** AU565 cells were pretreated with Geldanamycin (Gelda) at indicated dose for 24 hours and, subsequently, with trastuzumab (Trast) and docetaxel (Doc) alone or in combination, for 72 hours at indicated concentrations. Cell viability was assessed by Crystal Violet assay and results, expressed as percentage of cell growth relative to untreated control, represent the average ±SEM of three independent experiments. AU565 and AU565 shHER2 cells were treated with trastuzumab (Trast) and docetaxel (Doc), at indicated concentrations, for 72 hours. Cell viability was assessed by Crystal Violet assay and results, expressed as percentage of cell growth relative to untreated control, represent the average ±SEM of three independent experiments.

Supplementary Figure S5. Combination treatment between pertuzumab and docetaxel in HER2 context. AU565 and BT474 cells were treated with pertuzumab (Pert) and docetaxel (Doc), alone or in combination, for 72 hours at indicated concentrations. Cell viability was assessed by Crystal Violet assay and results, expressed as percentage of cell growth relative to untreated control, represent the average ±SEM of three independent experiments.

Supplementary Figure S6. HSP90 levels in representative HER2-driven breast cancer patients. Breast biopsies were subjected to immunohistochemistry (IHC) analyses to measure HSP90 levels. Results were quantified multiplying the percentage of positive neoplastic cells and staining intensity (1 (weak), 2 (moderate), 3 (strong). The images are representative of a negative (**A**) and positive (**B**) cases. 20X magnification (scale bar 200 μm).

Supplementary Figure S7. (**A**) Progression-free survival (PFS), Overall Survival (OS) and (**B**) Progression-free Survival Hormonal Treatment (PFS-HT) curves according to HSP90.

Supplementary Figure S8. Progression-free survival (PFS), Overall Survival (OS) curves according to HSP90 in the subgroup of first line treatment

Supplementary Figure S9. (**A**) Progression-free Survival Hormonal Treatment (PFS-HT) curves according to HSP90 in subgroup of first line treatment and (**B**) according to first line treatment in subgroup HSP90

Supplementary Figure S10. Progression-free survival (PFS) and Overall Survival (OS) curves of combined trastuzumab/pertuzumab treatment and trastuzumab monotherapy.
