# Supplementary material for: HER2-Driven Breast Cancer: Role of the Chaperonin HSP90 in Modulating Response to Trastuzumab-Based Therapeutic Combinations
Source: Int J Mol Sci. 2025 Jul 9;26(14):6593. doi: 10.3390/ijms26146593 (PMC12294885; doi:10.3390/ijms26146593)
Supplement: Supplementary file 1 [file ijms-26-06593-s001.zip › Supplementary Figures.pdf]

Table S1. Genetic status of breast cancer cell lines analyzed

Cell lines	HER2 status	Type	Morphology	Tumor origin	ATCC number	RRID Identificator	References
AU565	Amplificated	Adenocarcinoma	Epithelial	Pleural effusion	CRL-2351	CVCL_1074	[18,19]
BT474	Amplificated	Ductal carcinoma	Epithelial	Mammary gland	HTB-20	CVCL_VL01	[18,19]
SKBR3	Amplificated	Adenocarcinoma	Epithelial	Pleural effusion	HTB-30	CVCL_0033	[18,19]
MCF7	-	Adenocarcinoma	Epithelial	Pleural effusion	HTB-22	CVCL_0031	[18,19]
MDA-MB468	-	Adenocarcinoma	Epithelial	Pleural effusion	HTB-132	CVCL_0419	[18,19]
MDA-MB231	-	Adenocarcinoma	Epithelial	Pleural effusion	HTB-26	CVCL_0062	[18,19]
ZR75-1	-	Ductal carcinoma	Epithelial	Ascites	CRL-1500	CVCL_0588	[18,19]
BT549	-	Ductal carcinoma	Epithelial	Mammary gland	HTB-122	CVCL_1092	[17,19]
HCC1395	-	Ductal carcinoma	Epithelial	Mammary gland	CRL-2324	CVCL_1249	[17,19]
T47D	-	Ductal carcinoma	Epithelial	Pleural effusion	HTB-133	CVCL_0553	[17,19]
CALU3	Amplificated	Lung adenocarcinoma	Epithelial	Pleural effusion	HTB-55	CVCL_0609	[19]

Figure S1

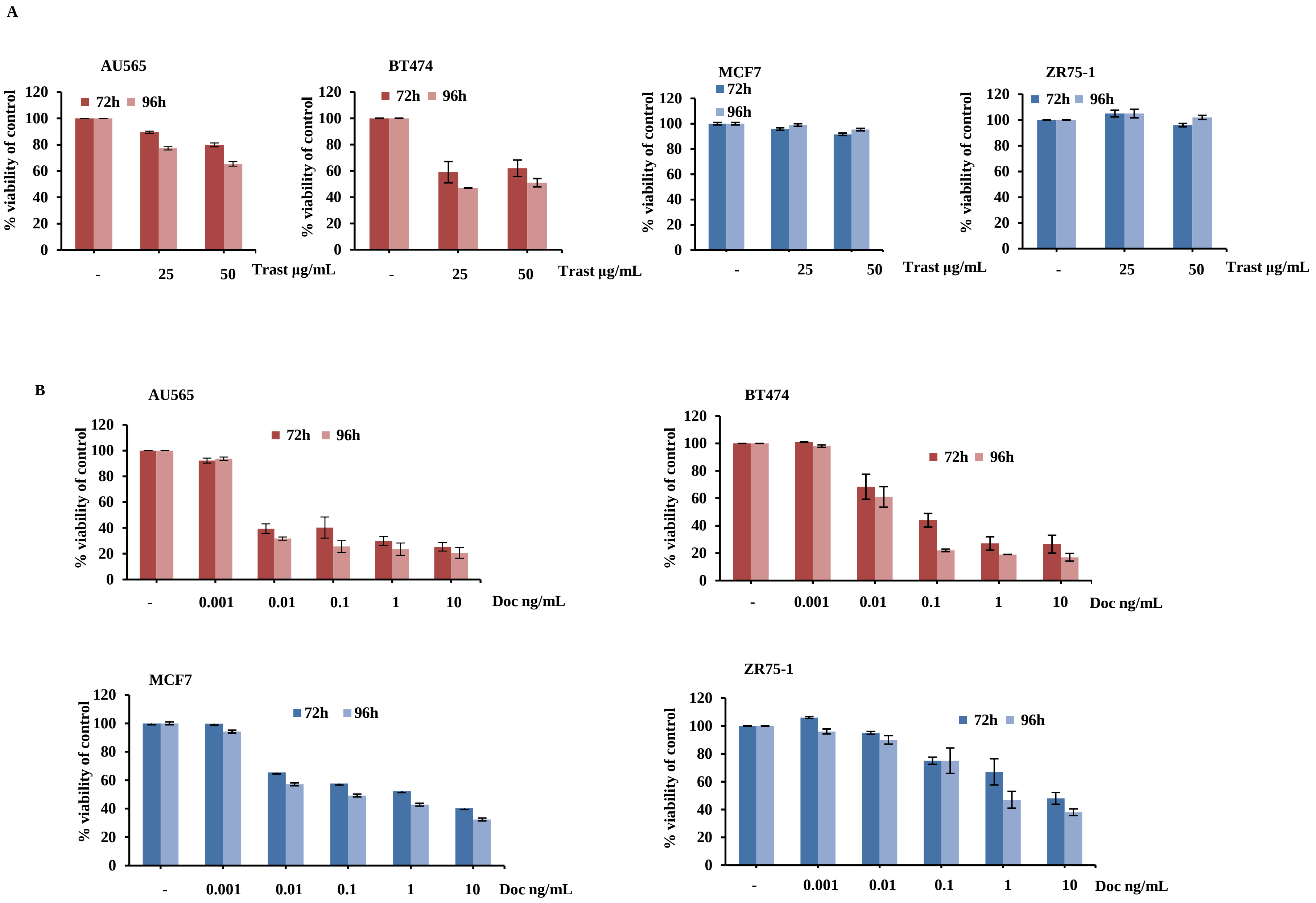
A
AU565
BT474
MCF7
ZR75-1
72h
96h
% viability of control
Trast µg/mL
B
Doc ng/mL
0.001
0.01
0.1
1
10

Table S2. Half maximal inhibitory concentration (IC50) of docetaxel treatment

Cell line	Docetaxel IC50 (ng/mL)	
	**72 hours**	**96 hours**
**AU565**	0.034	0.013
**BT474**	0.113	0.025
**MCF7**	0.72	0.305
**ZR75-1**	6.62	1.53

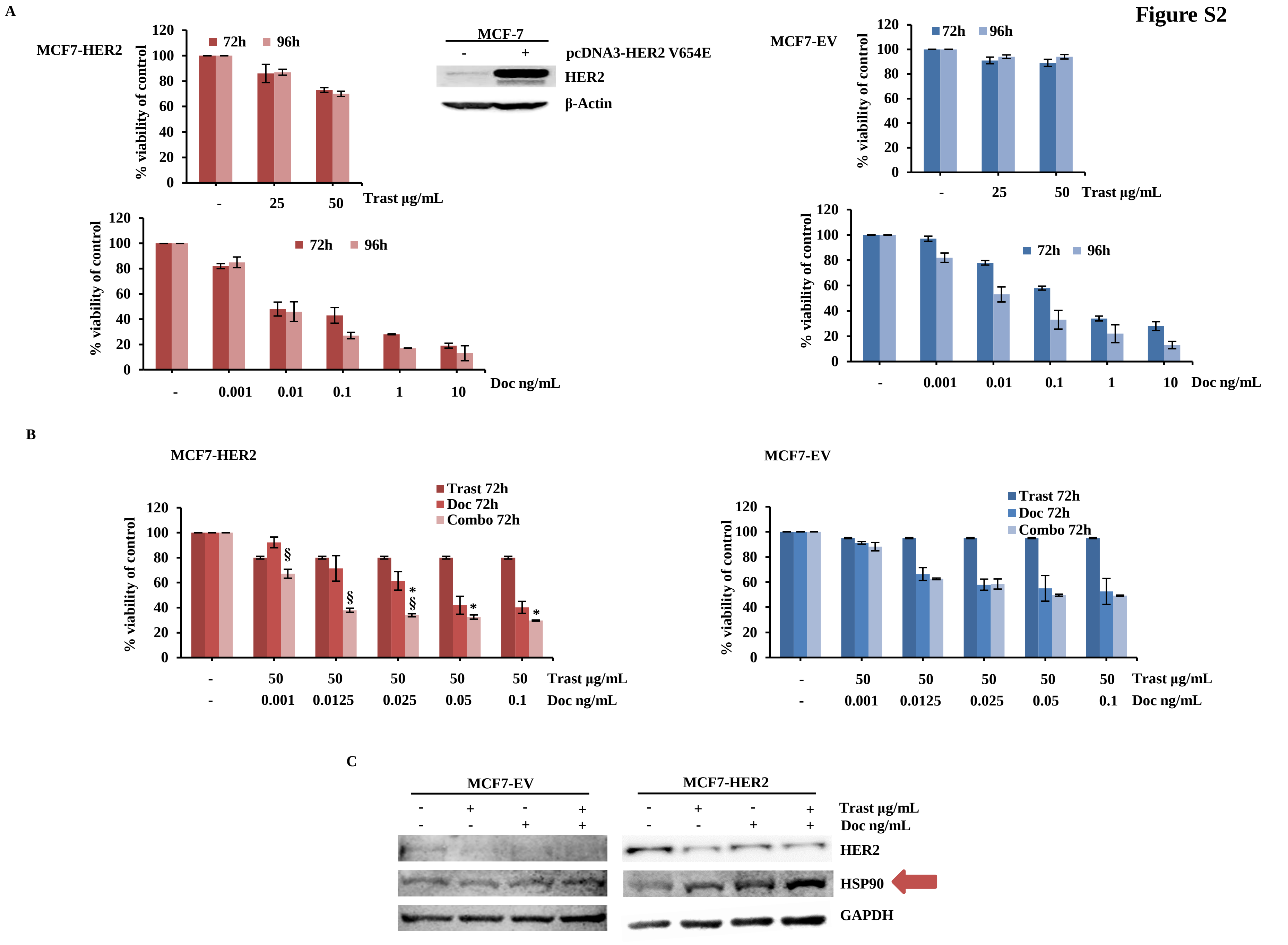

Figure S2
A
MCF7-HER2
72h
96h
% viability of control
Trast µg/mL
MCF-7
pcDNA3-HER2 V654E
HER2
β-Actin
MCF7-EV
Doc ng/mL
B
MCF7-HER2
Trast 72h
Doc 72h
Combo 72h
MCF7-EV
C
MCF7-EV
MCF7-HER2
HER2
HSP90
GAPDH

Table S3. Half maximal inhibitory concentration (IC50) of docetaxel treatment

**Cell line**	**Docetaxel IC50 (ng/mL)**	
	**72 hours**	**96 hours**
**MCF7-EV**	0.26	0.022
**MCF7-HER2**	0.032	0.014

Figure S3

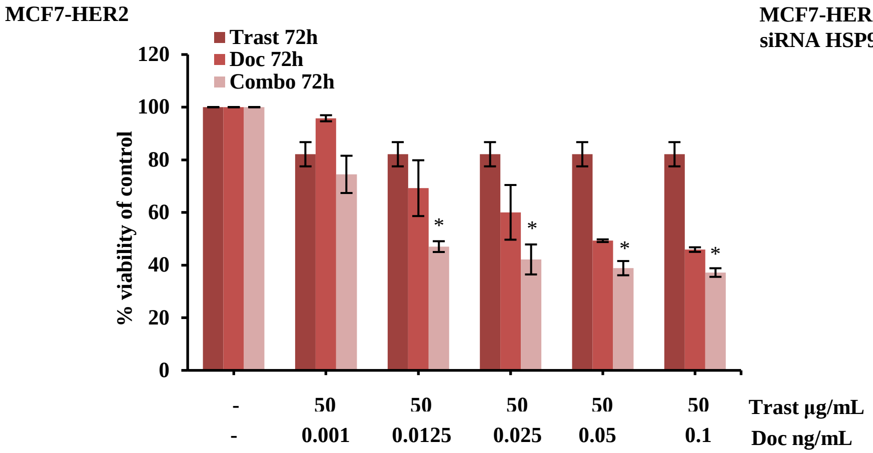
MCF7-HER2
Trast 72h
Doc 72h
Combo 72h
% viability of control
120
100
80
60
40
20
0
*
- 50 50 50 50 50 Trast µg/mL
- 0.001 0.0125 0.025 0.05 0.1 Doc ng/mL

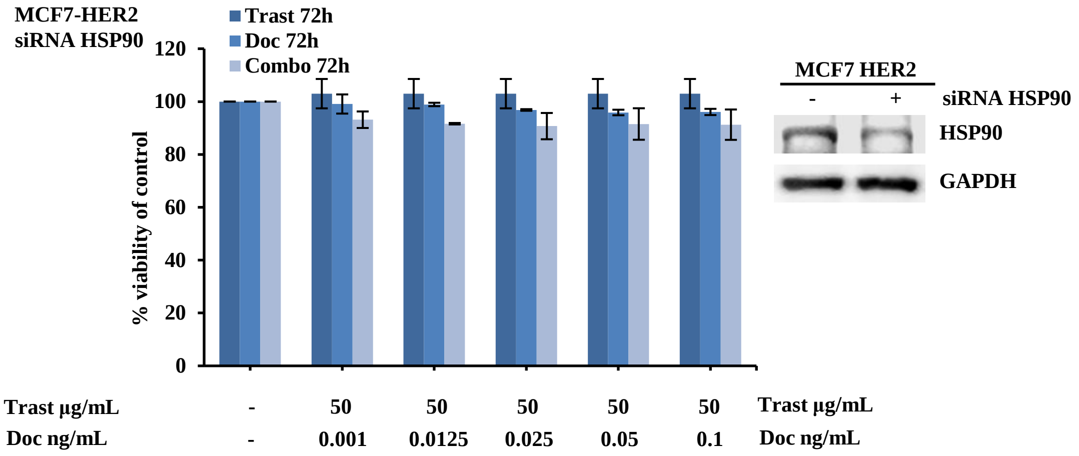
MCF7-HER2
siRNA HSP90
Trast 72h
Doc 72h
Combo 72h
% viability of control
120
100
80
60
40
20
0
- 50 50 50 50 50 Trast µg/mL
- 0.001 0.0125 0.025 0.05 0.1 Doc ng/mL
MCF7 HER2
- + siRNA HSP90
HSP90
GAPDH

Figure S4

A

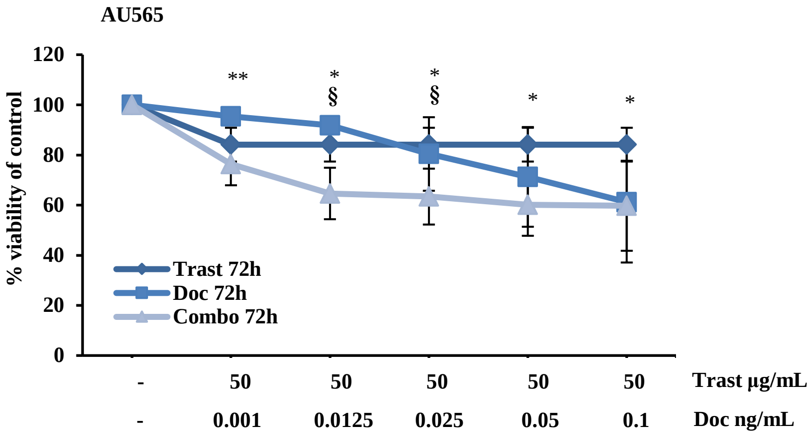
AU565
120
100
80
60
40
20
0
% viability of control
**
*
§
*
§
*
*
Trast 72h
Doc 72h
Combo 72h
- 50 50 50 50 50 Trast µg/mL
- 0.001 0.0125 0.025 0.05 0.1 Doc ng/mL

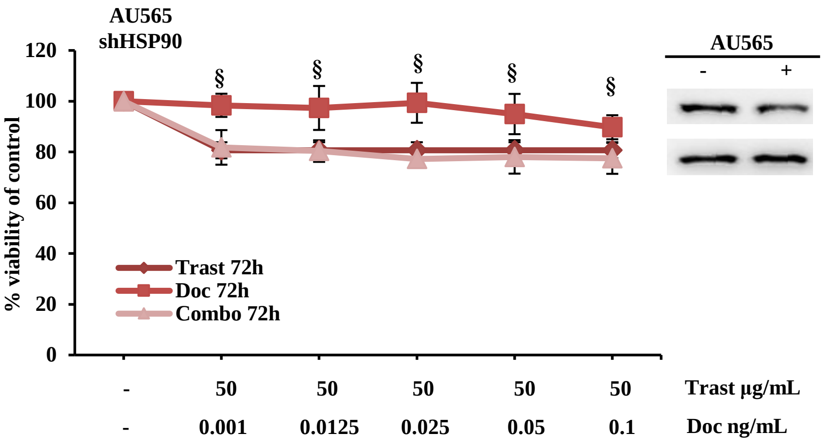
AU565
shHSP90
120
100
80
60
40
20
0
% viability of control
§
§
§
§
§
Trast 72h
Doc 72h
Combo 72h
- 50 50 50 50 50 Trast µg/mL
- 0.001 0.0125 0.025 0.05 0.1 Doc ng/mL

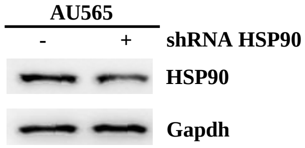
AU565
- +
shRNA HSP90
HSP90
Gapdh

B

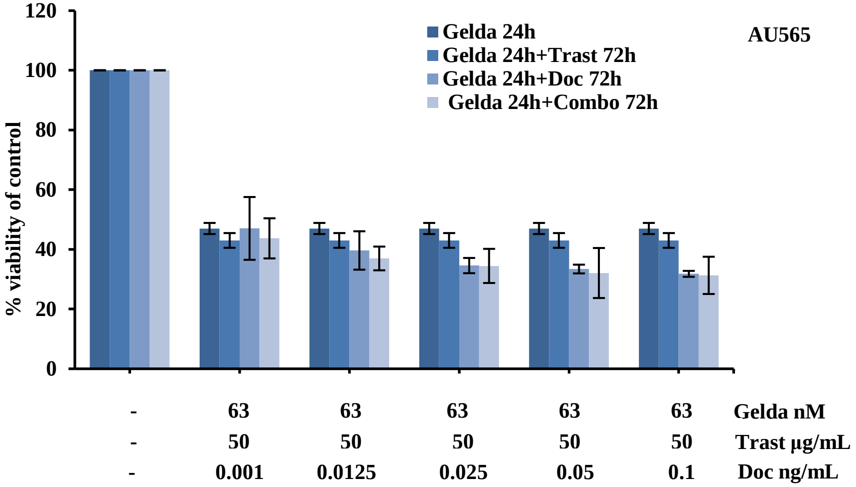
120
100
80
60
40
20
0
% viability of control
Gelda 24h
Gelda 24h+Trast 72h
Gelda 24h+Doc 72h
Gelda 24h+Combo 72h
AU565
- 63 63 63 63 63 Gelda nM
- 50 50 50 50 50 Trast µg/mL
- 0.001 0.0125 0.025 0.05 0.1 Doc ng/mL

Figure S5

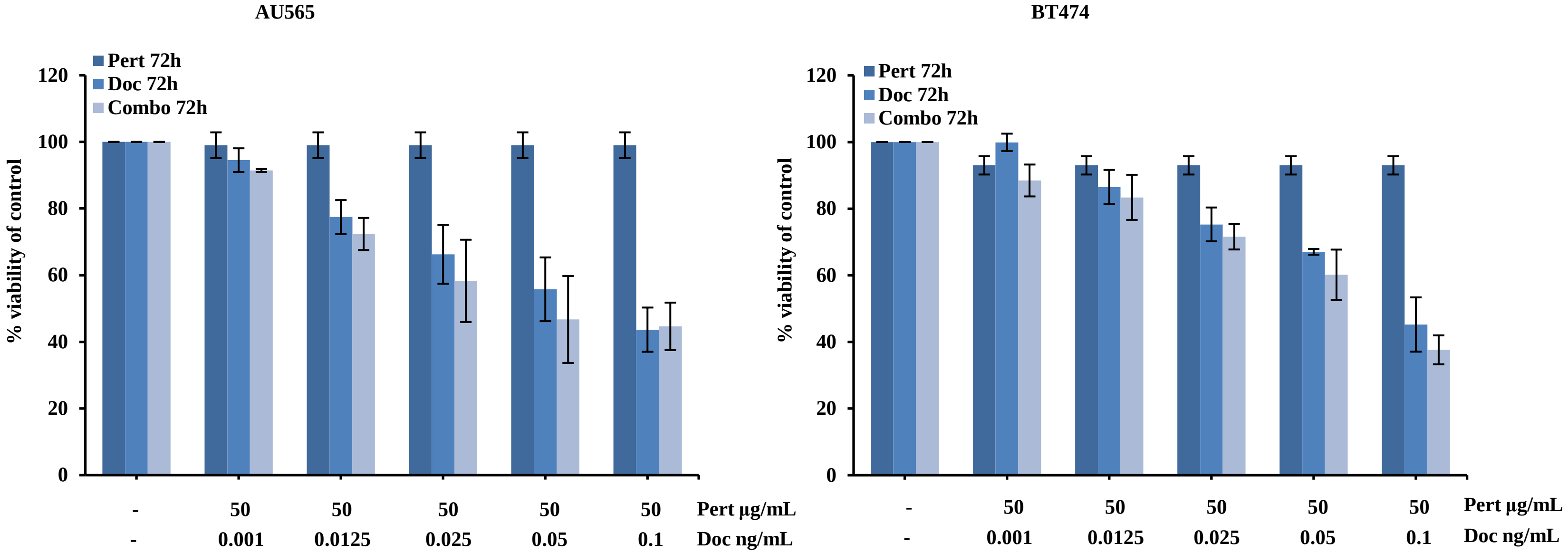
AU565
Pert 72h
Doc 72h
Combo 72h
% viability of control
0
20
40
60
80
100
120
- 50 50 50 50 50 Pert µg/mL
- 0.001 0.0125 0.025 0.05 0.1 Doc ng/mL
BT474
Pert 72h
Doc 72h
Combo 72h
% viability of control
0
20
40
60
80
100
120
- 50 50 50 50 50 Pert µg/mL
- 0.001 0.0125 0.025 0.05 0.1 Doc ng/mL

Table S4. Half maximal inhibitory concentration (IC50) of docetaxel treatment

TDM-1 IC50 (μg/mL)	
**AU565**	**AU565 siRNA HSP90**
34.9	3.15

Figure S6

HSP90 IHC

A **B**

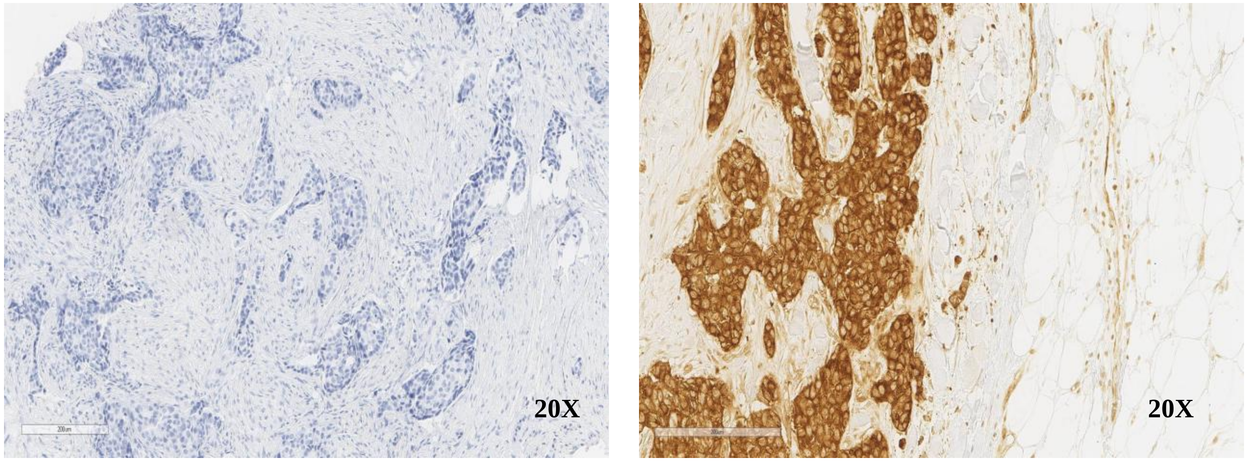

Figure S7

A

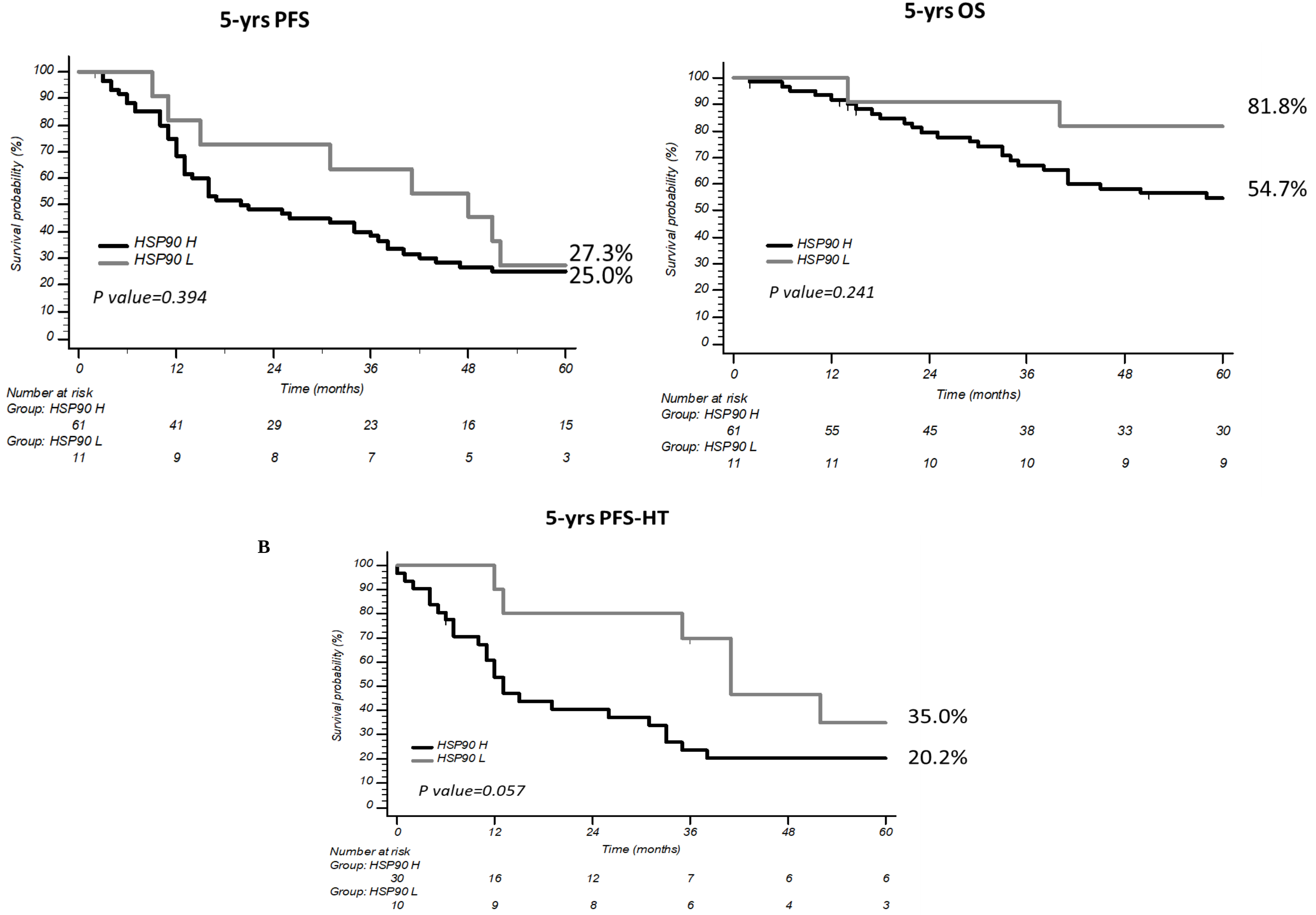
5-yrs PFS
Survival probability (%)
100
90
80
70
60
50
40
30
20
10
0
HSP90 H
HSP90 L
27.3%
25.0%
P value=0.394
0
12
24
36
48
60
Time (months)
Number at risk
Group: HSP90 H
61 41 29 23 16 15
Group: HSP90 L
11 9 8 7 5 3
5-yrs OS
Survival probability (%)
HSP90 H
HSP90 L
81.8%
54.7%
P value=0.241
Time (months)
Number at risk
Group: HSP90 H
61 55 45 38 33 30
Group: HSP90 L
11 11 10 10 9 9
5-yrs PFS-HT
B
Survival probability (%)
HSP90 H
HSP90 L
35.0%
20.2%
P value=0.057
Time (months)
Number at risk
Group: HSP90 H
30 16 12 7 6 6
Group: HSP90 L
10 9 8 6 4 3

Figure S8

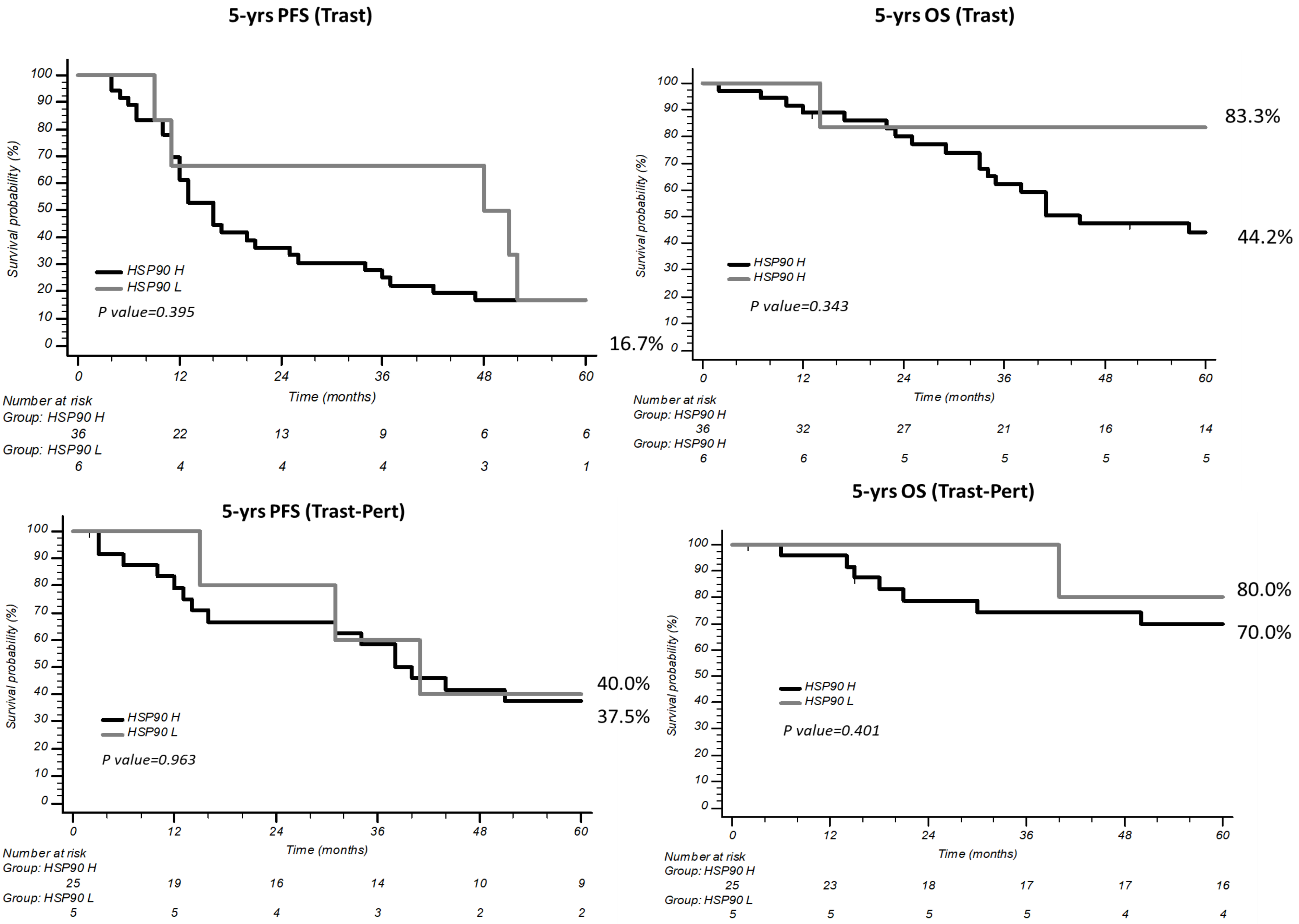

5-yrs PFS (Trast)
Survival probability (%)
Time (months)
HSP90 H
HSP90 L
P value=0.395
16.7%
Number at risk
Group: HSP90 H
36 22 13 9 6 6
Group: HSP90 L
6 4 4 4 3 1
5-yrs OS (Trast)
Survival probability (%)
Time (months)
HSP90 H
HSP90 H
P value=0.343
83.3%
44.2%
Number at risk
Group: HSP90 H
36 32 27 21 16 14
Group: HSP90 H
6 6 5 5 5 5
5-yrs PFS (Trast-Pert)
Survival probability (%)
Time (months)
HSP90 H
HSP90 L
P value=0.963
40.0%
37.5%
Number at risk
Group: HSP90 H
25 19 16 14 10 9
Group: HSP90 L
5 5 4 3 2 2
5-yrs OS (Trast-Pert)
Survival probability (%)
Time (months)
HSP90 H
HSP90 L
P value=0.401
80.0%
70.0%
Number at risk
Group: HSP90 H
25 23 18 17 17 16
Group: HSP90 L
5 5 5 5 4 4

Figure S9

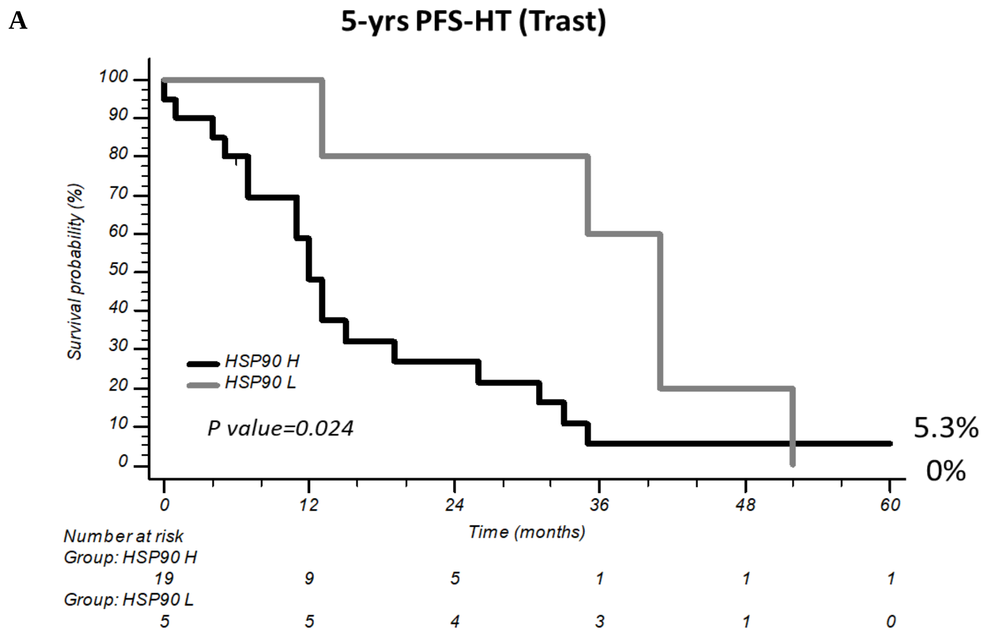
A
5-yrs PFS-HT (Trast)
Survival probability (%)
100
90
80
70
60
50
40
30
20
10
0
HSP90 H
HSP90 L
P value=0.024
5.3%
0%
0
12
24
36
48
60
Time (months)
Number at risk
Group: HSP90 H
19 9 5 1 1 1
Group: HSP90 L
5 5 4 3 1 0

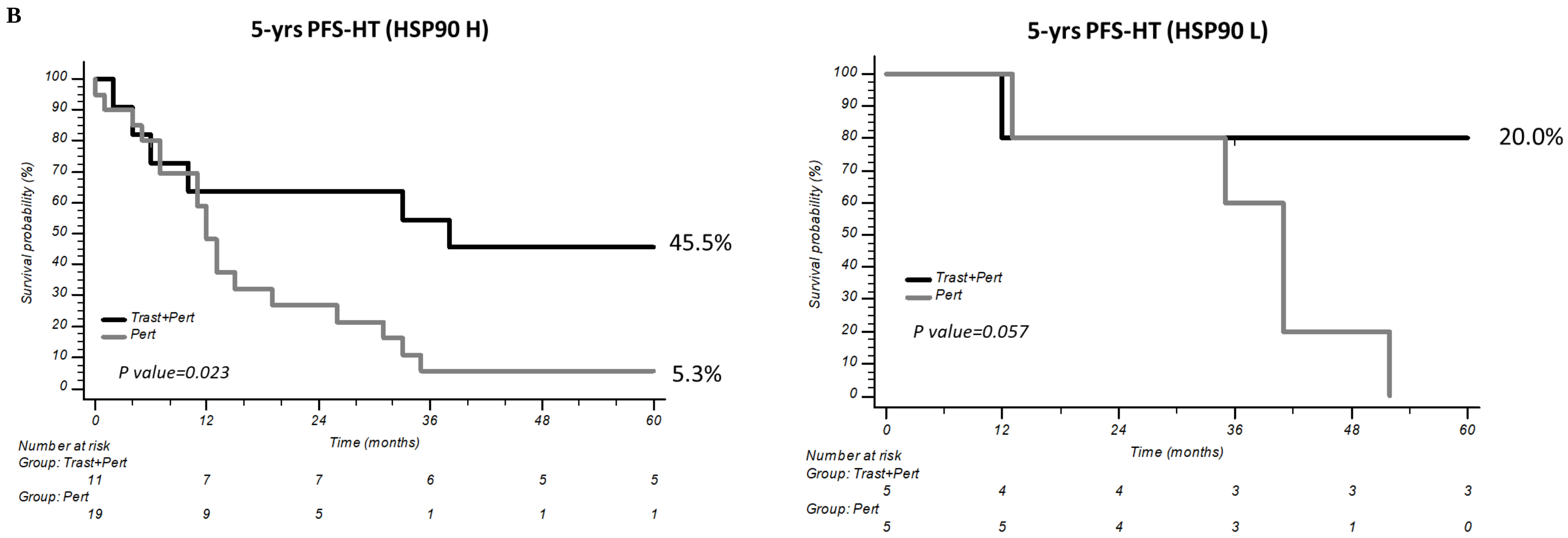
B
5-yrs PFS-HT (HSP90 H)
Survival probability (%)
Trast+Pert
Pert
P value=0.023
45.5%
5.3%
Time (months)
Number at risk
Group: Trast+Pert
11 7 7 6 5 5
Group: Pert
19 9 5 1 1 1
5-yrs PFS-HT (HSP90 L)
Survival probability (%)
Trast+Pert
Pert
P value=0.057
20.0%
Time (months)
Number at risk
Group: Trast+Pert
5 4 4 3 3 3
Group: Pert
5 5 4 3 1 0

Figure S10

PFS

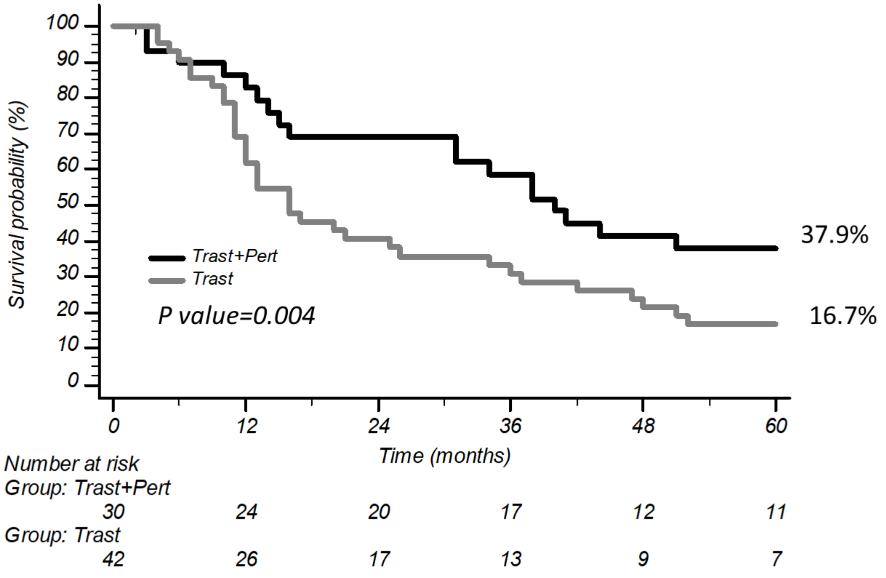

OS

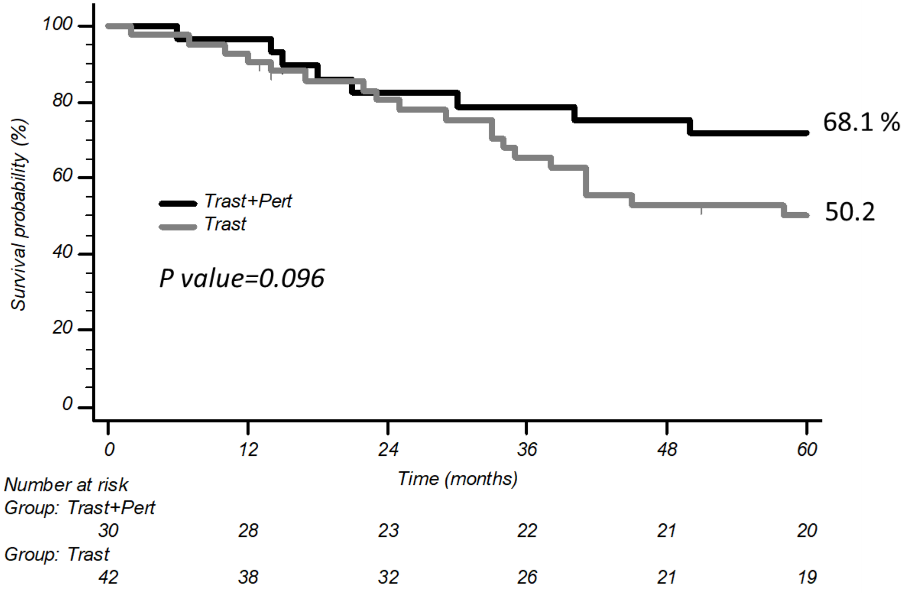

Table S5. Uni- and multi-variate analysis for OS from first-line treatment start.

**Variable**	**Comparison**	***Univariate***			***Multivariate***		
		**HR**	**95% CI**	***P*-value**	**HR**	**95% CI**	***P*-value**
HSP90 expression	High vs Low	1.673	0.700-3.998	0.247	-	-	-
Age	Continuous variable	1.021	0.998-1.045	0.078	**-**	-	-
First-line Treatment	Trastuzumab vs Trastuzumab + Pertuzumab	1.744	0.682-3.448	0.110	-	-	-
HT Maintenance	Yes vs No	1.095	0.669-2.110	0.785	-	-	-
Histology	Ductal vs Lobular vs Other	-	-	0.354	-	-	-
Surgery	Yes vs No	1.054	0.528-2.104	0.881	-	-	-
Metastatic *de novo*	No vs Yes	1.060	0.546-2.060	0.862	-	-	-
Grading	G3 vs G2	1.732	0.795-3.775	0.167	-	-	-
ER	Pos vs Neg	1.494	0.707-3.153	0.293	-	-	-
PGR	Pos vs Neg	1.527	0.780-2.986	0.217	-	-	-
Ki67	$\geq$20 vs <20	1.408	0.747-2.657	0.290	-	-	-
Visceral Met	No vs Yes	1.075	0.570-2.028	0.822	-	-	-
Bone-only Disease	Yes vs No	1.907	0.905-4.020	0.090	-	-	-
		Uni/multivariate					
		**HR**	**95% CI**	***P*-value**			
ECOG PS	***2 vs 0/1***	***5.409***	***2.406-12.153***	***<0.0001***			
